# An artificial habitat facilitates a climate-mediated range expansion into a suboptimal novel ecosystem

**DOI:** 10.1371/journal.pone.0211638

**Published:** 2019-02-20

**Authors:** Zachary J. Cannizzo, Blaine D. Griffen

**Affiliations:** 1 Marine Science Program, School of the Earth, Ocean, and Environment, University of South Carolina, Columbia, SC; 2 Department of Biology, Brigham Young University, Provo, UT; University of Potsdam, GERMANY

## Abstract

As the geographic ranges of tropical species and ecosystems continue to shift poleward with climate change, it is critical to prediction and management to identify factors that facilitate these expansions. This is especially true for range shifts that involve the decoupling of a shifting species from its historic ecosystem and the colonization of an ecosystem that it has not previously inhabited (i.e. is novel to the shifting species). In cases where the colonized ecosystem is suboptimal for the shifting species, stepping stone refuges may play a critical role in facilitating further expansion. Here we document the facilitation of the northward range expansion of the mangrove tree crab (*Aratus pisonii*) into the previously uninhabited salt marsh ecosystem by artificial boat docks. While the cold tolerance of crabs did not differ between habitats, they were found on docks 36 km and 22 km further north than elsewhere in the salt marsh after the winters of 2016-‘17 and ‘17-’18, respectively. This extended range-edge appears to be a result of docks within the salt marsh acting as a stepping stone refuge by providing this historically tropical species with a relatively warm thermal refuge during the winter that mitigates seasonal population die-backs exhibited elsewhere at the range-edge. Further, population abundances were higher on docks at the range-edge than in the surrounding salt marsh. While artificial habitats often favor the expansion of non-indigenous species, our results demonstrate the facilitation of a native species’ range shift into a suboptimal ecosystem which it has not previously inhabited. The potential for analogous and refuge habitats, artificial or otherwise, to increase the rate and success of range shifts could be critical to the fate of many current and future range shifting species.

## Introduction

One of the most conspicuous impacts of climate change is the worldwide shift in the geographic ranges of species and ecosystems [1–3]. In particular, many tropical species are expanding poleward into temperate regions [3,4]. The expansions of these species are often coupled with corresponding shifts of the foundation species, which structure a community and often provide the structural basis of an ecosystem (i.e. trees in a forest, coral on a reef, etc. *sensu* [5,6]), of their historic ecosystems [7]. However, differences in responses to changing environmental conditions lead some species to decouple their range shifts from those of their historic foundation species and expand at a faster rate [8]. This can result in shifting species colonizing ecosystems with which they have little or no ecological or evolutionary history [8]. These colonized ecosystems, which are novel to the expanding species, are likely to differ from the expanding species’ historic ecosystem in ways that will have consequences for its ecology, life history, and range shift.

Colonization of new ecosystems exposes species to novel biological and environmental interactions that may result in suboptimal conditions which slow further expansion [9–12]. However, pockets of habitat that provide improved conditions can act as stepping stone refuges allowing species to expand through otherwise unfavorable or uninhabitable habitat. Stepping stone refuges can take numerous forms from forest fragments [13] to artificial structures such as ponds [14,15] and gravel pits [16]. While stepping stone refuges are often thought of as corridors between favorable habitats [17–20], they may also increase the expansion into previously uninhabited ecosystems by providing refuge from suboptimal conditions that would otherwise limit the range. The geographic range of poleward-expanding species is often limited by winter temperatures [1,4]. While a species may expand northward during warmer months, winter die-backs are common at the range-edge, leading to a characteristic pattern of surges and setbacks [3,21,22]. For such species, habitats that provide thermal refuge may be critical to their ability to continue to expand poleward and have even been shown to nearly eliminate latitudinal gradients in thermal stress [23]. Even minimal thermal refuge could prevent the need to recolonize each year, allowing populations to establish further north than would otherwise be possible. For example, temperatures under the canopies of stunted mangrove stands in Louisiana are about 2 ^o^C warmer than the surrounding salt marsh leading to reduced frost damage and increased survival of recruiting mangrove seedlings [24,25]. Such a canopy-like feature is rare in the salt marsh but can also be found under artificial structures such as boat docks. If these structures similarly provide thermal refuge, they may permit poleward-expanding estuarine species to survive or persist further north than would otherwise be possible. Even a modest effect on the latitudinal expansion of the range of a species could be significant to its geographic coverage if it allows for expansion into a new region, such as a river system, or beyond a dispersal barrier.

The study of artificial habitats in range expansions often highlights their role in facilitating invasions [26–29] through their association with human activities such as shipping [27,30], their provision of bare substrate on which early successional invasive species can become established [27], or by being more similar to the native environment of the invader than the surrounding ecosystem [29]. However, these structures can also be beneficial to native species by acting as refuges and habitat analogues to their historic ecosystem [16,20,30] and may thus aid their range expansions. Analogous habitats, artificial habitats that resemble the historic ecosystem of a species within a suboptimal environment (*sensu* [31]), can provide a number of important ecological and life history benefits [12,32] and offer refuge from environmental impacts [12,33]. For example, boat docks act as a habitat analogue to the mangrove for the mangrove tree crab *Aratus pisonii* within colonized salt marshes [12]. This historically Neotropical mangrove associated crab [34–36] has recently outpaced the northern range expansion of mangroves and colonized salt marshes on the Southern US Atlantic coast, an ecosystem it has not previously inhabited and is thus novel to this species [37]. Crabs in the salt marsh experience inferior thermal and foraging conditions [12] and exhibit altered behavior [12,38], smaller body size and size at maturity [11,12], and reduced larval quality [11] compared to conspecifics in the mangrove. As such, the salt marsh is a suboptimal novel ecosystem for this native range expander [12]. However, docks within the salt marsh ecosystem mitigate many of these impacts by providing crabs with improved thermal and foraging conditions that result in increased size among other improvements to their life history and ecology [12]. Thus, if in addition to being a mangrove analogue, docks act as a stepping stone refuge and allow *A*. *pisonii* to expand northward more quickly, or survive further north than would otherwise be possible, they could play a critical role in determining the rate of this species’ expansion and its ultimate geographic extent.

Here we examine the impact of docks on the range expansion of *A*. *pisonii* through distributional surveys and measures relevant to individual and population survival: thermal differences between docks and salt marsh, *A*. *pisonii* cold tolerance, and relative population abundance. As the relatively short time since colonization and high gene flow from southern populations [39] likely preclude evolution of a higher cold tolerance in the salt marsh, we predict cold tolerance will not differ between habitats. However, we predict that docks will provide a critical thermal buffer during cold periods resulting in smaller winter die-backs, both in terms of relative abundance and geographical extent, than elsewhere at the range-edge leading to *A*. *pisonii* being found further north on docks within the salt marsh than in the salt marsh proper.

## Methods

### Study system

*Aratus pisonii* is a largely arboreal semi-terrestrial crab historically found in mangrove forests throughout the neotropics [34,35]. After a short larval dispersal phase (~20 days [35]), these crabs settle on structure as immature adults and live for two to three years [35]. The ecology of this crab has historically been closely tied to mangrove trees. Crabs move only short distances from central foraging areas centered around individual trees to which they show site fidelity [38] and utilize fresh leaves of the red mangrove *Rhizophora mangle* as their primary food source [36]. Due to high aquatic predation, *A*. *pisonii* actively avoid water, except to wet gills or release larvae, by climbing any available structure on the rising tide, even leaving shelter to do so ([35,40] ZJ Cannizzo pers. observ.).

The colonization of the salt marsh ecosystem has had numerous impacts on this crab’s ecology. Individuals in the salt marsh exhibit a smaller maximum and average body size [11,12], as well as reduced reproductive potential [11] compared to conspecifics in the mangrove. These changes appear to be driven by suboptimal conditions in the salt marsh including an inferior diet and a suboptmially warm summer thermal habitat [12]. However, crabs found on boat docks within the salt marsh experience cooler summer temperatures resulting from the shaded habitat and sturdy, vertical structure that is more similar to the mangrove than the surrounding salt marsh [12]. In combination with an improved diet deriving from fouling communities [12], docks allow the crabs found there to be similar in size to conspecifics in the mangrove while exhibiting less dangerous behavior among other ecological and life history benefits [12]. Docks even appear to partially mitigate disturbance impacts [33]. Thus, by providing conditions more similar to the mangrove than the surrounding colonized ecosystem, docks act as both a mangrove analogue and a refuge habitat for *A*. *pisonii* within the suboptimal novel ecosystem of the salt marsh [12].

### Distributional surveys

To determine the northern extent of the range of *A*. *pisonii* in the salt marsh and on docks, and the extent to which those distributions changed after winter die-backs, we conducted distributional surveys at the northern edge of the crab’s range in the autumns (November 2016, 2017) and following springs (May 2017, June 2018) of consecutive years. As *A*. *pisonii* in this region largely stop reproducing in October (ZJ Cannizzo unpublished work), this allowed us to record the furthest Northward extent of the species each year. The most recent survey of the geographical range of *A*. *pisonii* was undertaken in 2013 [37] and cited the northern extent as Little Satilla Creek, Georgia (31^o^5’32”N) with no individuals found just south at Jekyll Island, Georgia (31^o^2’31”N). Thus, we began our autumn 2016 survey at Jekyll Island and moved north along the coast until we encountered two consecutive sites where no *A*. *pisonii* were found (Table 1). Sites were selected based on accessibility, access to both salt marsh and dock habitat via kayak, and were as similar to each other as possible. Further, the salt marsh explored at each site was either across the river or at least 0.75 km from the nearest dock. Given that adults rarely stray more than 25 m from a central foraging area [38], this ensured that crabs found in the salt marsh had no interaction with docks. In all habitats, the largely terrestrial *A*. *pisonii* climbs structure to avoid rising waters and aquatic predation (ZJ Cannizzo pers. observ. [35,40]). Therefore, we always conducted surveys during tidal inundation of the salt marsh to increase the likelihood that if *A*. *pisonii* were present, they would be found climbing marsh grasses. Sites were explored for at least one hour before they were designated as having no *A*. *pisonii*.

**Table 1:** Site locations, presence (Y) and absence (N) of *A*. *pisonii* and ovigerous/mature females (denoted in parentheses), presence of *A*. *pisonii* in 2013 survey (Riley et al., 2014 [37]). Asterisks denote sites of thermal logger deployment.—denotes that the site was not sampled during the given survey. No females were captured in the marsh at Big Talbot State Park in the spring of 2018.

Site	Lat.-Long.	Salt marsh autumn 2016	Salt marsh Spring 2017 (Ovigerous/Mature)	Dock autumn 2016	Dock Spring 2017 (Ovigerous/Mature)	Salt marsh autumn 2017	Salt marsh Spring 2018 (Ovigerous/Mature)	Dock autumn 2017	Dock Spring 2018 (Ovigerous/Mature)	Riley et al.
		2016–2017 Survey Year				2017–2018 Survey Year				
Sunbury Boat Ramp	31^o^45’51”N 81^o^16’41”W	—	—	—	—	N	N	N	N	—
Halfmoon Marina	31^o^41’42”N 81^o^16’17”W	—	—	—	—	N	N	N	N	—
Barbour River	31^o^37’17”N 81^o^15’49”W	N	N	N	N	Y	N	Y	N	—
Dallas Bluff *	31^o^35’25”N 81^o^18’8”W	N	N	N	N	N	N	Y	N	—
Belleville Launch	31^o^31’52”N 81^o^21’32”	N	N	Y	Y (N/N)	N	N	Y	N	—
Sapelo Island NERR *	31^o^27’13”N 81^o^21’46”W	Y	N	Y	Y (N/Y)	Y	N	Y	N	—
Blue N. Hall Landing	31^o^24’21”N 81^o^23’33”W	Y	N	Y	Y (Y/Y)	Y	N	Y	N	—
Village Creek	31^o^12’19”N 81^o^21’36”W	—	Y (Y/Y)	—	Y (Y/Y)	Y	N	Y	N	—
Little Satilla River *	31^o^5’32”N 81^o^34’15”W	Y	N	Y	Y (Y/Y)	Y	N	Y	N	Y
Jekyll Island *	31^o^2’31”N 81^o^25’21”W	Y	Y (Y/Y)	Y	Y (Y/Y)	Y	N	Y	Y (N/Y)	N
Crooked River	30^o^50’44”N 81^o^33’34”W	—	—	—	—	—	Y (N/Y)	—	Y (Y/Y)	Y
Fernandina Beach	30^o^40’16”N 81^o^27’56”W	—	—	—	—	—	Y (N/Y)	—	Y (Y/Y)	Y
Big Talbot State Park	30^o^22’30”N 81^o^35’6”W	—	—	—	—	—	Y	—	Y (Y/Y)	Y

While the presence-absence of individuals is an important measure of the geographic extent of a species, the establishment of a reproductive population is of particular importance to its long-term persistence in a newly colonized location. Thus, we chose to conduct the spring surveys during the week before the full moon of May 2017 and the week before the new moon of June 2018. This allowed us to take advantage of the lunar synchronization of *A*. *pisonii* reproduction [35] by conducting surveys during, or shortly after, the first reproductive cycle of the breeding season (ZJ Cannizzo unpublished work) ensuring that any individuals encountered had overwintered at the sites where they were found. While logistical constraints caused a delay in the spring 2018 survey, the survey was conducted less than 2 weeks after the first reproductive cycle. Given the ~20-day planktonic stage of *A*. *pisonii* larvae [35], any crabs found were unlikely to have been recent recruits. Further, we did not encounter any crabs below reproductive size in this survey ensuring that the observed crabs had survived the winter at the site where they were found. We conducted the spring surveys using the same methods as the autumn surveys and noted the presence or absence of ovigerous (egg carrying) females at each site.

During the first survey year (2016–2017), we captured 15 crabs, or all that were found, at each site and, before release, recorded the sex and size (measured as carapace width to the nearest 0.1mm) of each individual. For the spring surveys, size data were compared to the smallest and average sizes of ovigerous females recorded from each habitat in and around St. Augustine, FL (29^o^ 52’N-30^o^ 8’N), where *A*. *pisonii* has established populations (Salt marsh: Smallest = 8.0, Avg. = 12.2±1.6; Docks: Smallest = 11.1, Avg. = 17.0±2.2) (ZJ Cannizzo unpublished work). A similar strategy was employed in the second survey year (2017–2018) with catch effort added to gain a measure of relative abundance (see below).

During each survey after the autumn of 2016, we sequentially added additional sites to more accurately pin-point the northern location of *A*. *pisonii*. This resulted in the addition of Village Creek in the spring of 2017, Halfmoon Marina and Sunbury Boat Ramp in the autumn of 2017, and three sites in the spring of 2018: Big Talbot Island, Fernandina Beach, and Crooked River (Table 1).

### Relative abundance

During the second survey year (2017–2018), we sought to measure the relative abundance of *A*. *pisonii* in each habitat at each site as catch per unit effort (CPUE) in crabs caught per minute. As many sites provided access to only a single dock, CPUE on docks was always examined on the first dock we could access at all sites that contained crabs. This differs from presence/absence (see above) where all accessible docks were explored. Before being released, the sex and size of each captured crab was also recorded as described above. This measure of relative abundance was intended to allow for both a relative measure of the reduction of abundance after a winter die-back and the identification of an expansion front. In spring 2018, we also explored relative population abundances in three dock (Palm Valley: 30^o^07’57”N, Yacht Club: 29^o^53’09”N, Vilano Inlet: 29^o^56’33”N) and three salt marsh sites (GTM NERR: 30^o^0’49”N, Anastasia State Park: 29^o^52’40”N, Vilano Inlet: 29^o^56’33”N) that have been inhabited by *A*. *pisonii* for more than a decade allowing for a comparison of abundance in edge and established populations. We explored relative population abundance (CPUE) using a mixed-effects linear model with latitude, habitat (salt marsh/dock), and season (spring/autumn) as explanatory variables including site as a random effect. As established populations were not sampled in the autumn, a similar model, including the variable of level of establishment (edge/established), was also used for data from the spring survey only to compare edge and established populations.

### Cold tolerance

To fully understand the extent to which docks may act as a thermal refuge, we sought to determine the cold tolerance of *A*. *pisonii*. We collected 30 crabs from each habitat where *A*. *pisonii* is found (salt marsh, docks in the salt marsh, mangrove). Crabs from the mangrove were included in this analysis to determine if there has been any change in the cold tolerance of *A*. *pisonii* due to colonization of the salt marsh ecosystem. As this study was conducted just after the unusually cold winter of 2017–2018 (see below), we collected crabs from established populations to prevent doing undue damage to the highly impacted populations at the range-edge and to avoid biasing the results with any unusually cold-tolerant individuals that had survived the extreme cold conditions. Crabs were collected from sites representative of the habitat being explored. The mangrove was represented by crabs collected from Pepper Park (27^o^29’42”N), the salt marsh by crabs from Anastasia State Park (29^o^52’40”N), and docks by crabs from the St. Augustine Yacht Club (29^o^53’09”N). It is possible that populations from the extreme range-edge have higher cold tolerances than we record here. However, the short time since *A*. *pisonii* has colonized these northernmost sites (<10 years, ZJ Cannizzo pers. comm. with local residents) and the high gene flow between habitats [39] suggest little time for such populations to evolve a significantly higher cold tolerance. Nevertheless, our results should be considered a benchmark against which we can compare measures of temperature to infer biologically relevant extremes rather than true cold tolerances of each population.

Upon collection, we determined the size and sex of each individual before placing it in a plastic aquarium (22.8x15.2x16.5 cm, l x w x h) with food (fresh *Rhizophora mangle* leaves) and a petri dish of water inside an incubator maintained at a 12:12 light-dark cycle for the duration of the experiment. Water was changed every other day with fresh food given *ad libitum*. While crabs do not have access to mangrove leaves in the salt marsh and dock habitats, leaves were given to all crabs to avoid any confounding effects of food type when determining cold tolerance. Crabs were allowed to acclimate to incubator conditions at 25 ^o^C for 48 hours after which the temperature was linearly and gradually decreased to 20 ^o^C over a 12-hour period. This, and all other changes in temperature, were achieved through manual programing of the Percival (Perry, Iowa, USA) E36L1X incubator in which the experiment took place. After 36 hours, the temperature was again decreased linearly over 12 hours to 15 ^o^C. Following a further 36-hour acclimation period, the experimental program was initiated. 15 ^o^C was chosen as a starting point as this is a temperature regularly experienced by crabs in the Florida mangrove ecosystem and preliminary experiments showed no mortality at this temperature. As terrestrial organisms often experience short nightly bursts of cold temperatures with warming during the day, which could be critical to poikilothermic organisms such as *A*. *pisonii*, we created a temperature program that mimicked a daily cooling and warming cycle. This program began at the beginning of the dark cycle with an 11-hour linear decrease to the target temperature. The target temperature was then held for one hour until the beginning of the light cycle at which time the temperature was increased linearly to 15 ^o^C over a 6-hour period, where it was maintained until the next dark cycle. The target temperature on the first night was set to 14 ^o^C and was decreased by 1 ^o^C with each subsequent night. Crab mortality was checked each day after the program had leveled to 15 ^o^C. The progressive lowering of minimum temperature allowed for a methodologically rigorous determination of cold-tolerance while the daily warming allowed crabs the potential chance to recover from extreme cold, similar to a daily warming cycle. Once all crabs had died, we corrected the temperatures crabs experienced with data gathered from thermal loggers placed throughout the incubator during the experiment (to account for slight spatial differences in temperature within the incubator). We then examined cold-tolerance using a cox proportional hazards model with habitat, sex, and crab size as explanatory variables for the number of days survived. The proportional hazards assumption was met as Schoenfeld residuals were independent of time both globally and for all covariates (p>0.10). We further determined the median lethal temperature (LT50) and complete lethal temperature (LT100) of *A*. *pisonii* overall and for each habitat.

### Temperature measurements

To determine if docks provide a thermal refuge during the winter, we placed 4 Onset (Bourne, Massachusetts, USA) HOBO thermal data loggers (2 under a dock and 2 in the salt marsh less than 0.5 km away) at each of 4 sites (Table 1) that spanned the autumn 2017 range-edge. In the salt marsh, loggers were placed on PVC piping even with the top of marsh grasses to avoid inundation during high tides and reflect the height at which crabs are found during high tide periods. Similarly, loggers under docks were placed in an area that remained out of water during high tide to reflect conditions crabs experience. Loggers recorded temperature simultaneously every 10 minutes from December 11, 2017 to April 30, 2018. The average of the two loggers deployed in each habitat was used to calculate the temperature for that habitat at that site at 10-minute intervals. We then used habitat type and site location (in degrees latitude) as explanatory variables in a linear model to explore their effects on the number of days with at least one continuous hour below *A*. *pisonii* LT50 and LT100 (separate models). Similar models were used to explore the effects of habitat and latitude on the total time, in hours, spent below each threshold. In addition, we determined the daily minimum temperature recorded in each habitat at each site and employed a liner model to determine if latitude and habitat impacted the average minimum temperature experienced over the duration of the deployment. Water temperature data were also retrieved from USGS climate station 22035975, Hudson Creek, which is located at the Sapelo Island site.

While the loggers were deployed, the southeastern United States experienced an unusually cold winter. Thus, we retrieved 1988–2018 temperature data from the University of Georgia Marine Institute on Sapelo Island, Georgia, located only 10 km from the Sapelo Island site. To determine if the winter of 2017–2018 truly represented an extreme cold event, we adapted the definition of an extreme event from Canning-Clode and Carlton [22] as a period of five consecutive days with the minimum temperature below the 10^th^ percentile of daily minimum temperatures drawn from a baseline of the past 30 winters (December-March). We also used the dataset to determine if there were any particularly extreme events where the minimum temperature remained below the 5^th^ percentile for five consecutive days.

### Ethics statement

Crabs used for the cold tolerance experiment were collected under Florida Department of Environmental Protection permit #07101720. Permission for data logger deployment was obtained from the Georgia Department of Natural Resources and the owners of private docks. All private land was accessed with land-owner permission while protected land was accessed with permission from the Georgia Department of Natural Resources and the Guana Tolomato Matanzas National Estuarine Research Reserve. No protected species were sampled.

## Results

### Distributional surveys

**Fig 1:**
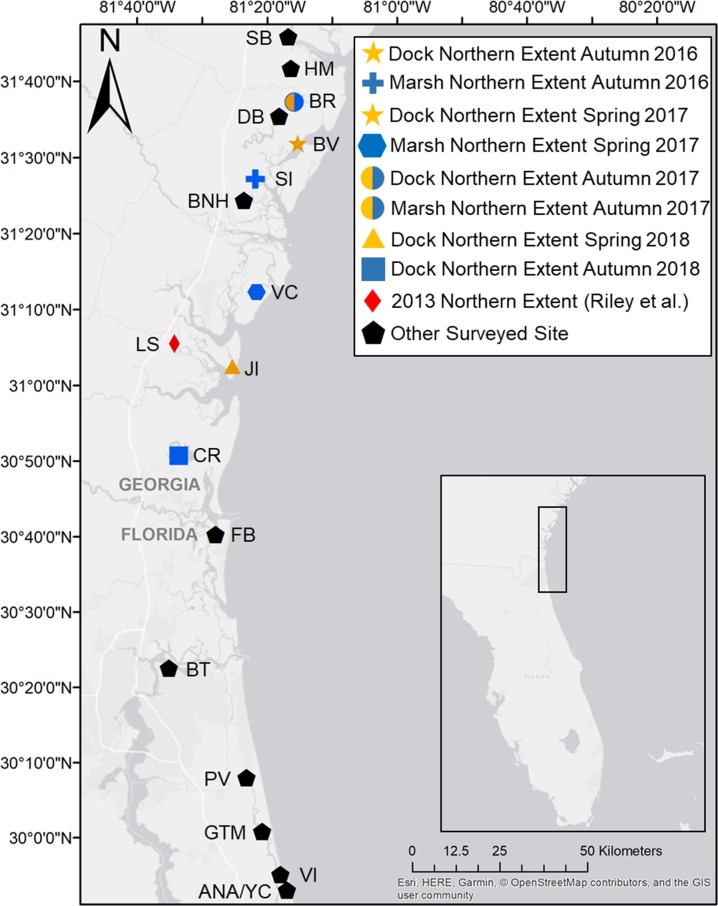
Map of survey sites along the Southeast Atlantic Bight. Locations of northern extents are delineated by color. Blue symbols correspond to salt marsh northern extents while orange symbols correspond to dock northern extents. Red symbol corresponds to northern extent as of 2013. Black symbols represent sites that were surveyed but did not represent a northern geographic extent of *A*. *pisonii* in any survey. Top to bottom: SB = Sunbury Boat Ramp, HM = Half Moon Marina, BR = Barbour River, DB = Dallas Bluff, BV = Belleville Launch, SI = Sapelo Island NERR, BNH = Blue N Hall Landing, VC = Village Creek, LS = Little Satilla Creek, JI = Jekyll Island, CR = Crooked River, FB = Fernandina Beach, BT = Big Talbot State Park, PV = Palm Valley, GTM = GTM NERR, VI = Vilano Inlet, ANA/YC = Anastasia State Park/Yacht Club. Both dock and salt marsh habitat were surveyed at all sites with the exception of PV (dock only) and GTM (marsh only). Inset displays map location. Figure was built upon base map data republished from OpenStreetMap under a CC BY license, with permission from the OpenStreetMap Foundation, original copyright 2010.

In the autumn 2016 survey, *A*. *pisonii* were found 4.65 minutes of latitude (~9 km) further north on docks (Belleville) than in the salt marsh (Sapelo Island, Table 1, Fig 1). Further, *A*. *pisonii* were found 21.68 minutes of latitude (~40 km) further north than in the 2013 survey [37] (Little Satilla, Table 1, Fig 1). The following spring 2017 survey revealed that the range of *A*. *pisonii* in the salt marsh (Village Creek) had contracted south 14.90 minutes of latitude (~28 km) over the winter (Table 1, Fig 1). However, there was no change in the range of *A*. *pisonii* on docks. Thus, there was a 19.55 minute of latitude (~36 km) difference between the northernmost established population of *A*. *pisonii* on docks and in the salt marsh. Further, during the spring survey ovigerous females were found in the salt marsh at all sites where crabs where found, while ovigerous females on docks were found at all but the two northernmost sites (Belleville and Sapelo Island, Table 1), both sites where no crabs were found in the marsh. While no ovigerous females were found on the docks at Sapelo Island NERR, there were a number of females large enough to be mature and were thus likely reproductive but not ovigerous at the time of survey due to the relatively low reproductive activity of *A*. *pisonii* in May (ZJ Cannizzo unpublished work).

The autumn 2017 survey revealed that *A*. *pisonii* had expanded northward 5.41 minutes of latitude (~10 km) from the previous northernmost location during the summer; 31.75 minutes of latitude (~59 km) further north than the 2013 survey [37] (Table 1, Fig 1). While numerous crabs were found on docks at this location (Barbour River), only one juvenile male (7.5 mm) was found in the salt marsh, likely representing a recent colonization. The nearest location where we found a mature crab in the salt marsh was 10.07 minutes of latitude (~19 km) to the south (Sapelo Island, Table 1). Between the autumn 2017 and spring 2018 surveys, the Southeastern US experienced one of the coldest winters of the past 30 years (see below). This coincided with an extreme die-back of *A*. *pisonii* with the northern extent retreating 34.76 minutes of latitude (~64 km) on docks (Jekyll Island) and 46.56 minutes of latitude (~86 km) in the salt marsh (Crooked River) resulting in a loss of 3.01 and 14.80 minutes of latitude from docks and salt marsh respectively from the range-edge recorded in 2013 [37] (Table 1, Fig 1). However, *A*. *pisonii* were still found 11.79 minutes of latitude (~22 km) further north on docks than in the salt marsh proper.

The current northernmost non-seasonal extent of *A*. *pisonii* is Jekyll Island (31^o^2’31”N; Table 1) with two individuals found on a dock in the spring of 2018. Using the 1918 northern extent of Miami (25^o^48’N) [34], we can update the rate of *A*. *pisonii* range expansion to 58 km/decade, which is slower than both the 72 km/decade average rate of marine range expansions [41] and the previous estimate of 62 km/decade for this species [37]. Further, the estimate, if calculated from the 2016–2017 survey alone, would have been 64 km/decade highlighting the importance of encompassing extreme events and setbacks when determining rates of geographic range shifts.

As different sites were examined during each survey, statistical analyses of crab size was not possible. However, consistent with previous studies [12], the average sizes of crabs were consistently higher on docks than in the salt marsh (Table 2). The average sizes of crabs were also lower each spring than during the previous fall in both habitats (Table 2).

**Table 2:** The average and range of sizes of all crabs and ovigerous females examined from each habitat during each survey. —denotes that no ovigerous females were found during the survey.

Habitat	Season-year	Body size (Avg. ± SD mm)	Body size range (mm)	Ovigerous female size (Avg. ±SD, mm)	Ovigerous female size range (mm)
Dock	Autumn 2016	15.3±4.4	7.6–22.8	—	—
Salt marsh	Autumn 2016	9.8±2.7	6.4–16.8	—	—
Dock	Spring 2017	14.1±2.6	10.0–22.9	16.0±1.3	15.0–17.8
Salt marsh	Spring 2017	11.8±1.3	9.1–14.8	12.2±1.0	11.5–12.9
Dock	Autumn 2017	16.0±3.9	7.2–23.4	—	—
Salt marsh	Autumn 2017	10.8±1.8	7.0–13.7	—	—
Dock	Spring 2018	15.1±2.5	9.2–20.1	15.7±2.1	11.4–19.0
Salt marsh	Autumn 2018	12.4±1.8	6.4–17	12.3±1.4	10.0–16.6

### Relative abundance

**Fig 2:**
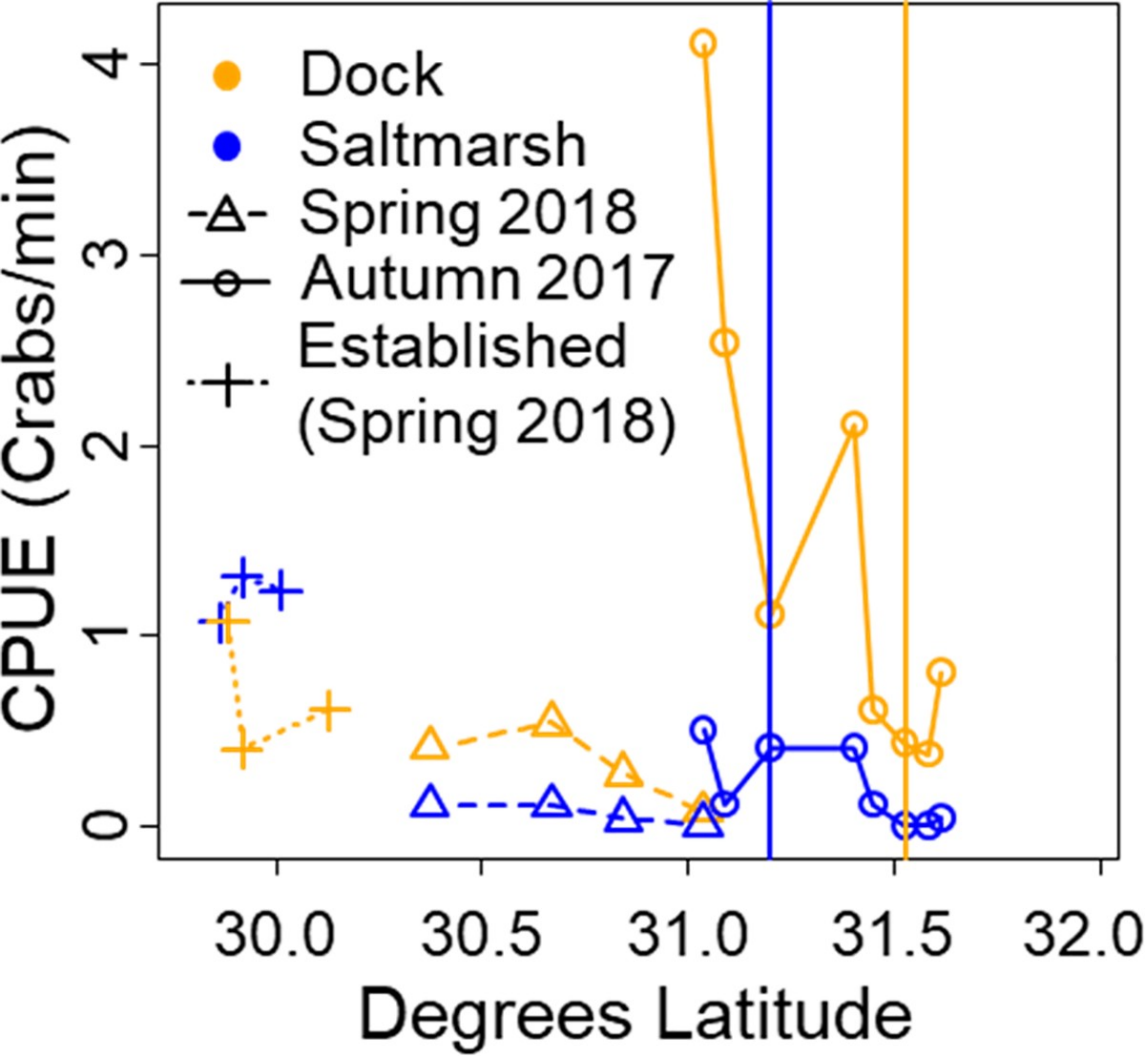
*A*. *pisonii* relative abundance as catch per unit effort (CPUE) in the salt marsh and on docks of sites surveyed during the 2017–2018 surveys and three sites per habitat in the established range, surveyed Spring 2018. Vertical blue and orange lines represent the northern extent of *A*. *pisonii* as of the Spring 2017 survey in the salt marsh and dock habitats respectively. Lack of autumn 2017 data south of 31^o^ N signifies no sampling of these sites during the autumn 2017 survey. CPUE data was not collected Spring 2017.

The relative abundance (CPUE) of *A*. *pisonii* was higher on docks than in the nearby salt marsh (LMER: z_40_ = -2.400, estim. = -0.459, p = 0.021; Fig 2) and decreased both from autumn to spring (LMER: z_40_ = -4.236, estim. = -0.921, p<0.001) and with increasing latitude (LMER: z_40_ = -3.735, estim. = -0.7149, p<0.001). The relative abundances were also higher in the established populations of both habitats during the spring survey (LM: z_24_ = -3.703, estim. = -0.568, p = 0.001; Fig 2). While the drastic die-back of *A*. *pisonii* prevented direct comparisons of abundance between the autumn and spring in individual sites, the one site where crabs were found on docks in both surveys (Jekyll Island) experienced a greater than 61-fold decrease in abundance, highlighting the devastating impact of the extreme winter among even those populations that were not eliminated.

### Cold tolerance

**Fig 3:**
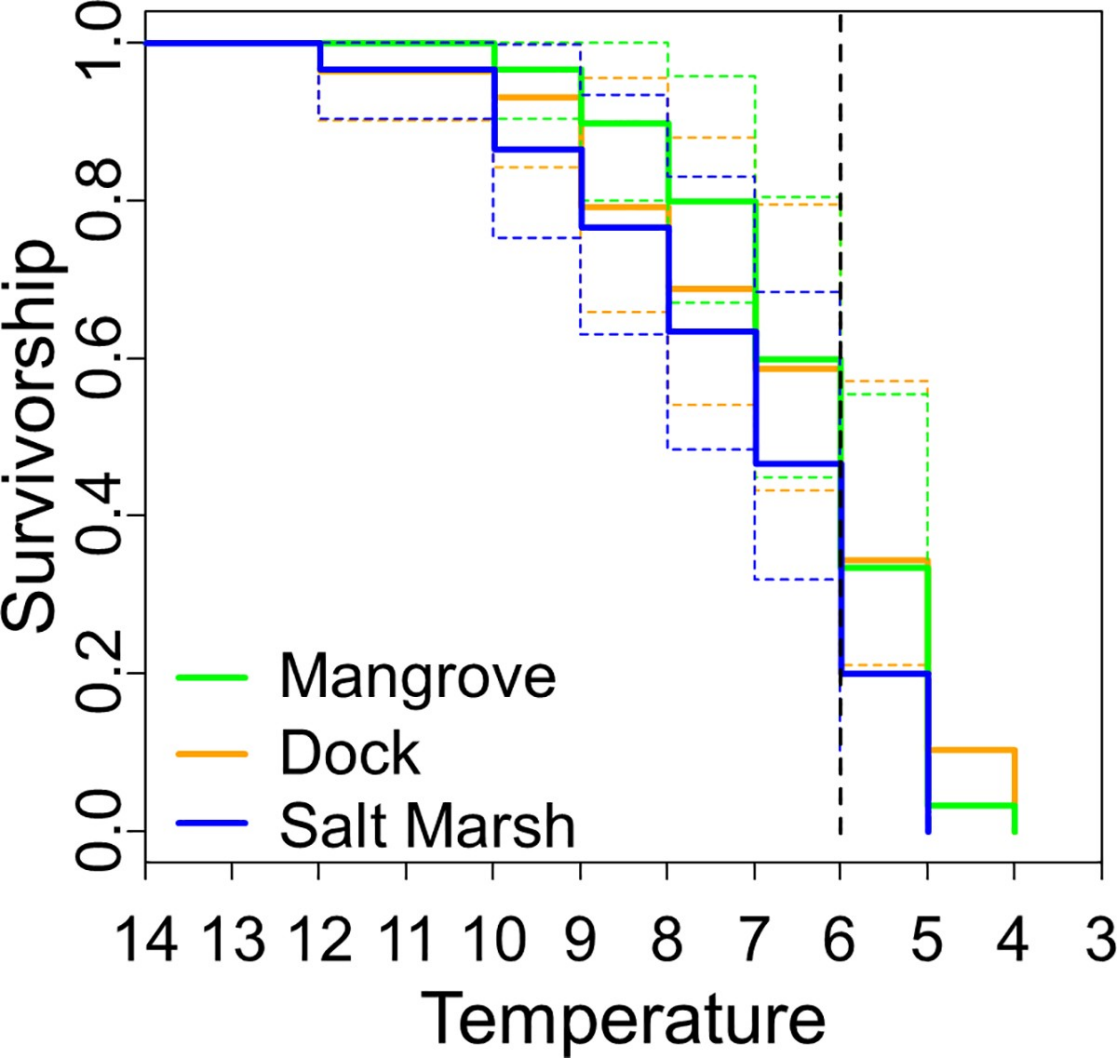
Kaplan-Meier curves comparing cold tolerances of *A*. *pisonii* from different habitats. Colored dashes lines represent 95% confidence intervals for each habitat. Vertical black-dashed line represents LT50.

The overall LT50 for *A*. *pisonii* was 6 ^o^C with an LT100 of 4 ^o^C. When habitats were examined independently, these values were the same for crabs from the mangrove and dock habitats but slightly warmer for crabs from the salt marsh (LT50 = 7 ^o^C, LT100 = 5 ^o^C). Despite the slightly warmer lethal temperatures for salt marsh crabs, there was no effect of habitat on crab survival (Cox PH: dock vs. mangrove: z = 0.414, p = 0.679; dock vs. salt marsh: z = 1.741, p = 0.082; mangrove vs. salt marsh: z = 1.513, p = 0.130; Fig 3), which was also independent of sex (Cox PH: z = 0.776, p = 0.438) and size (Cox PH: z = 0.597, p = 0.551).

### Temperature measurements

**Fig 4:**
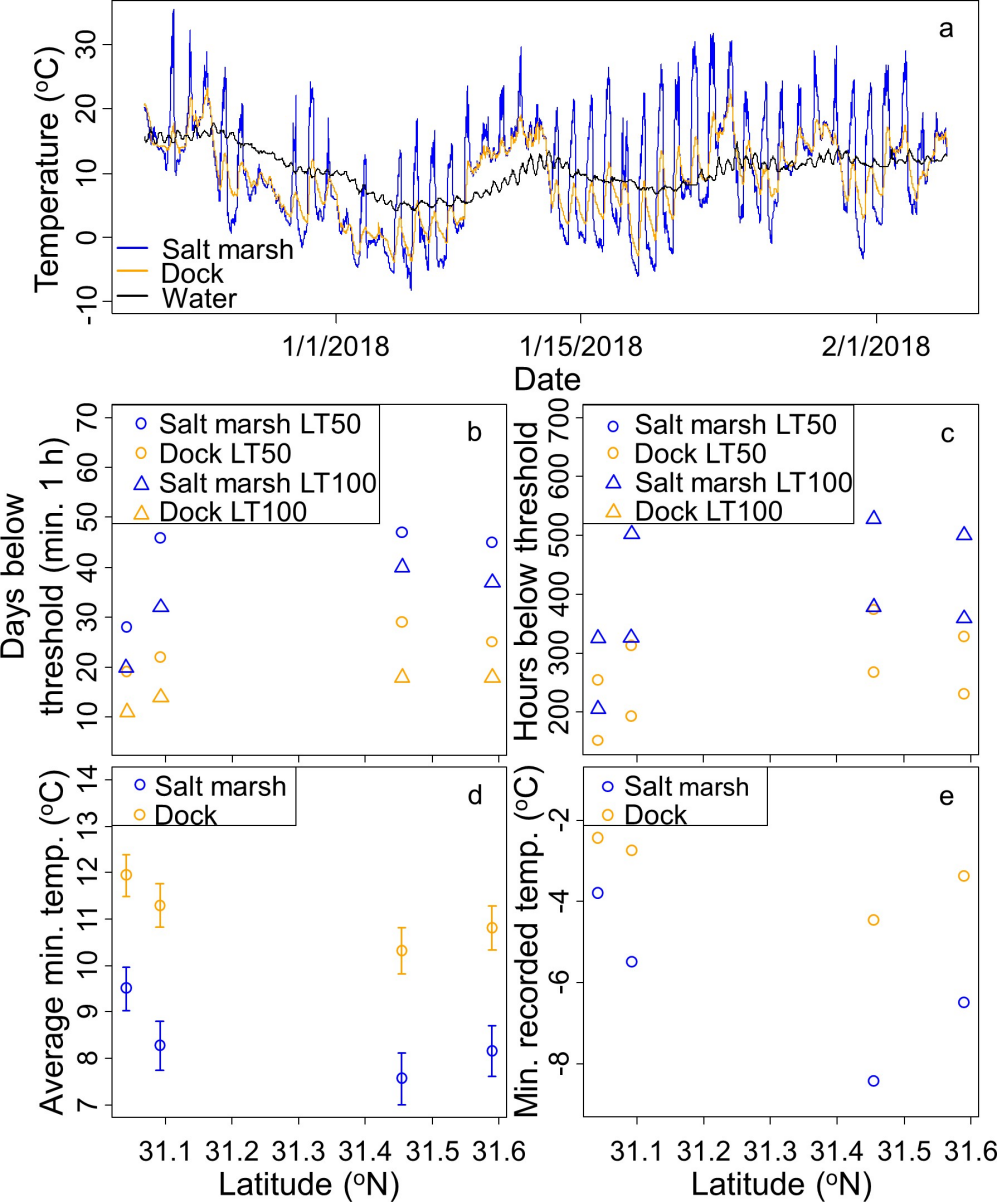
(A) Temperatures under a dock and in a nearby salt marsh at Sapelo Island. Water temperatures from a nearby climate station. (B) Days where the temperature remained below *A*. *pisonii* LT50 and LT100 for at least one continuous hour across habitats and sites where thermal loggers were deployed. (C) Total hours below *A*. *pisonii* LT50 and LT100 for at least one continuous hour across habitats and sites. (D) Minimum daily temperatures (Mean±SE) recorded over the deployment period across habitats and sites. (E) Absolute minimum temperature recorded in each site/habitat.

Nighttime temperatures under docks were constantly 2–5 ^o^C warmer than in the nearby salt marsh (Fig 4A) but did drop below *A*. *pisonii* cold tolerance at even the southernmost site where loggers were deployed (Fig 4). Docks also appear to generally act as a temperature buffer exhibiting lower daytime temperatures and less extreme temperature swings than in the salt marsh proper (Fig 4A). In addition, the water temperature at Sapelo Island was often warmer than the nightly air temperatures experienced in either the salt marsh or dock habitats (Fig 4A).

Compared to the dock habitat, the salt marsh experienced more days where the temperature stayed below both the LT50 and LT100 of *A*. *pisonii* for at least an hour (LM: LT50: z_5_ = 4.165 estim. = 17.750, p = 0.009; LT100: z_5_ = 5.202, estim. = 17.000, p = 0.003; Fig 4B). Further, while sites further north experienced more days under the LT100 threshold (LM: z_5_ = 2.743, estim. = 19.219, p = 0.041; Fig 4B), the latitudinal location of sites did not impact the number of days below the LT50 threshold (LM: z_5_ = 1.804, estim. = 16.480, p = 0.131; Fig 4B). Similarly, the total time spent below LT50 and LT100 was higher in the salt marsh (LM: LT50: z_5_ = 3.314, estim. = 146.38, p = 0.021; LT100: z_5_ = 3.382, estim. = 106.88, p = 0.020; Fig 4C). However, only the total time under LT100 increased with latitude (LM: LT50: z_5_ = 1.881, estim. = 178.11, p = 0.119; LT100: z_5_ = 2.794, estim. = 189.32, p = 0.0383 Fig 4C). In addition to the lethal temperature thresholds, the daily minimums were both colder in the salt marsh (LM: z_1125_ = -7.681, estim. = -2.718, p<0.001; Fig 4D and 4E) and decreased with increasing latitude (LM: z_1125_ = -2.781, estim. = -2.109, p = 0.006; Fig 4D and 4E).

The 10^th^ percentile of minimum winter temperatures, based on a baseline of the previous 30 winters retrieved from the Sapelo Island climate station (see above), was 0.6 ^o^C while the 5^th^ percentile was -1.7 ^o^C. Using these values, we determined that the winter of 2017–2018 represented a cold extreme with an extreme event (at least five consecutive days with minimums below the 10^th^ percentile) from January 1–7 during which a particularly extreme event (at least five consecutive days with minimums below the 5^th^ percentile) also occurred from January 2–6.

## Discussion

We have shown that an artificial habitat facilitates the range shift of a native species into a natural ecosystem that it has not previously inhabited. This anthropogenic facilitation of a range shift differs from that usually seen in species invasions where species are often first actively transported to a new geographic location (ex. through ballast water or bait buckets) before they can expand under their own power. In contrast, *A*. *pisonii* has expanded out of its historic range largely without direct anthropogenic aid. This is not an entirely unknown phenomenon as artificial feeders and urban rubble have been shown to facilitate the range expansions of Anna’s humming bird and the black redstart respectively [42,43]. However, unlike in the *A*. *pisonii* range expansion, these birds did not colonize ecosystems that were ecologically and evolutionarily novel to the species, but merely expanded their geographic and seasonal extents within previously inhabited ecosystems.

Extreme cold events are known to cause setbacks to the range expansions of tropical species, a phenomenon that has even been described for an aquatic invasive crab (the green porcelain crab *Petrolithses armatus*) that is undergoing a similar range expansion up the southeast US Atlantic coast [3]. However, we have shown that artificial habitats can play an important role in mitigating these impacts. Our results suggest that the artificial dock habitat acts as a stepping stone refuge by allowing *A*. *pisonii* to establish populations further north and expand more rapidly into the colonized salt marsh ecosystem than would otherwise be possible. By acting as a thermal refuge, docks minimize the winter die-back of seasonal populations that occurs elsewhere in the salt marsh ecosystem. The 2–5 ^o^C warmer conditions under docks during cold nights can be the difference between life and death, as seen in other tropical range expanding species such as mangroves [25], providing the thermal refuge that *A*. *pisonii* needs to survive and potentially expanding the geographical extent of the climatic envelope of the species. Recognizing this ability of artificial habitats to expand climatic envelopes (i.e. alter the physiological tolerability of an environment) could be critical to the accurate parameterization of mechanistic species distribution models used to predict range shift outcomes [44].

Despite the warmer conditions under docks, temperatures did drop below the apparent cold-tolerance of *A*. *pisonii* in both the dock and marsh, particularly during the extreme cold event from January 1–7, 2018. Yet, in the dock habitat some individuals survived at the southernmost site where thermal loggers were deployed. The survivors may have sheltered in microhabitats that were warmed during the day or retreated into the water, which stayed above the thermal minimum of *A*. *pisonii*. The ability to retreat to warmer water is another possible benefit provided by docks as most remain partially submerged throughout the tidal cycle. In contrast, crabs facing extreme cold during low-tide in the salt marsh are left with few if any thermal refuges (though they could feasibly utilize fiddler crab burrows). If crabs do find thermal shelter in the water, a strategy used to limit overheating in the summer [12], they could still experience increased mortality as aquatic predation on *A*. *pisonii* is high, particularly in the salt marsh [45]. Thus, even if crabs in the marsh can retreat to warmer water, the warmer aerial conditions under docks likely force this retreat less often, reducing secondary impacts of predation.

By providing thermal refuge during the critical cool nights of winter, docks have the potential to act as a stepping stone refuge and increase the rate of *A*. *pisonii* geographic expansion over what would otherwise be possible. Simply the ability to survive further north on docks increases the penetration of this species’ range into estuaries and river systems where it would not otherwise be found. Ovigerous females, or at least females of reproductive size, were found on docks at all sites in spring 2018 and all but the furthest northward site in spring 2017. Docks therefore prevent the need to reestablish every spring by providing a stock of reproductively mature crabs. As *A*. *pisonii* depends on larval dispersal and produces more, higher quality larvae on docks (ZJ Cannizzo unpublished work), the ability of the population to reproduce at sites further north has the potential to accelerate the range expansion of this species. However, even if mature individuals survive the winter, they must find a mate. While *A*. *pisonii* relative abundance decreased with latitude and fell during the winter in both habitats, it was consistently lower in the salt marsh in range-edge populations. Thus, even if some individuals survive extreme cold events in the salt marsh, the population may face an Allee effect until re-colonization from docks or more southern populations can replenish the breeding stock. While there has been no direct study of Allee effects in this species, they are a common problem faced by range-edge populations ([46] and references therein). In contrast, whether a result of the differing structural nature of the habitats, the thermal refuge docks provide, or some other factor such as reduced predation on docks, populations on docks are more densely populated, potentially reducing any post-winter Allee effect. This higher abundance on docks could be in part due to docks, as a single structure, concentrating crabs, whereas crabs are more diffuse in the salt marsh. However, if this is the case, it is an inherent, ecologically relevant difference between these habitats. Further, the higher relative abundance in the salt marsh in established regions suggests that the higher CPUE on docks at the range-edge is not simply a result of a sampling artifact. This reversal from higher relative abundances in the salt marsh in the established range to the dock at the range-edge may reflect either greater die-backs in range-edge marshes or preferential establishment on docks. Either would result in docks decreasing potential Allee effects and increasing expansion and colonization.

Ultimately, this work represents a little-studied aspect of range shift ecology: the ability of an artificial habitat analogue to act as a stepping stone refuge and accelerate the rate of a range shift, or at least the geographical penetration, of a native species into an ecosystem to which it is ecologically and evolutionarily naїve. There have been several discussions of the use of artificial habitats or habitat modifications to minimize the impacts of climate change on species in their historic ecosystems [47,48]. However, while many of these proposals focus on creating more favorable microhabitats within altered historic ecosystems [48–50], there has been little discussion of the use of artificial habitats to provide refuge for native range shifting species in novel ecosystems (but see [17,33]). While the role of artificial habitats as stepping stones for invasive species has garnered discussion [26,27,29], the shift in focus to their potential as refuge for native range shifters is a subtle but managerially important distinction. There has been robust discussion of the use of corridors and stepping stones to aid range shifts between favorable habitats [51,52], but their role in facilitating penetration into novel ecosystems has largely been contained to discussions of species invasions [26,27,29]. In fact, the discussion of anthropogenic habitats within range shift ecology largely focuses on the impediment they impose to native shifting species [53–55]. However, this study highlights the role that artificial structures can play in facilitating the range expansions of native species. In fact, artificial structures can provide critical refuge habitat that not only increases the permeability of the habitat matrix during range shifts but may accelerate the range shift itself.

As the number of species shifting their geographic ranges increases, deciphering the factors that impact shifting rates will be critical to understanding, predicting and managing outcomes. Artificial habitats have the potential to provide refuge from suboptimal novel conditions allowing species to shift more rapidly and more deeply into colonized ecosystems than would otherwise be possible. Thus, this study supplements work on expansions of non-indigenous species by highlighting the critical role that artificial stepping stone habitats can play in the range expansions of native species into novel ecosystems. Ultimately, the potential of analogous and refuge habitats, artificial or otherwise, to increase the rate and success of range shifts could be critical to the fate of many current and future range shifting species.
